# Triglyceride–Glucose Index and Extracellular Volume Fraction in Patients With Heart Failure

**DOI:** 10.3389/fcvm.2021.704462

**Published:** 2021-06-17

**Authors:** Shaomin Yang, Yongxing Du, Ziwei Liu, Rong Zhang, Xiaoxin Lin, Yufeng Ouyang, Haixiong Chen

**Affiliations:** Department of Radiology, Shunde Hospital, Southern Medical University, Foshan, China

**Keywords:** triglyceride-glucose index, heart failure, insulin resistance, biomarkers, myocardial fibrosis

## Abstract

**Background:** The triglyceride–glucose (TyG) index had been proposed as a reliable surrogate marker of insulin resistance. We aimed to evaluate the association between TyG index and myocardial fibrosis, which was quantified by extracellular volume (ECV) fraction using cardiovascular magnetic resonance (CMR) examination, and their prognostic value in patients with heart failure (HF).

**Methods:** In this retrospective cohort study, 103 hospitalized HF patients were included. ECV fraction was calculated using CMR measurements and T1 mapping. TyG index was calculated using fasting triglyceride and blood glucose. The primary outcome events were defined as all-cause mortality and HF hospitalization during follow-up.

**Results:** During the median follow-up of 12.3 months, 39 patients (37.9%) experienced primary outcome events and had higher levels of TyG index, N-terminal pro–B-type natriuretic peptide (NT-proBNP), and ECV fraction compared with those without events. Multivariate linear regression analysis showed that the TyG index was the significant factor determined for ECV fraction (*r*_partial_ = 0.36, *P* = 0.01). In multivariate Cox regression analysis, presence of diabetes [hazard ratio (HR) = 1.28, 95% confidence interval (CI) = 1.01–1.62], higher TyG index (HR = 2.01, 95% CI = 1.03–4.01), ECV fraction (HR = 1.73, 95% CI = 1.04–2.88), and NT-proBNP (HR = 2.13, 95% CI = 1.08–4.20) were independent risk factors for the primary outcome events.

**Conclusions:** TyG index is a novel biomarker of myocardial fibrosis in HF patients and can be considered as a useful risk stratification metric in the management of HF.

## Introduction

Epidemiological studies have shown that heart failure (HF) is a growing global public health burden, with prevalence up to 1 to 2% in the adult population (1). Detection of novel biomarkers and mechanisms would be of significant clinical importance for the prevention and treatment of HF (2, 3). It had been documented that metabolic disorders, including obesity, insulin resistance, and diabetes mellitus (DM) play an important role in the development and progression of HF (4). The triglyceride–glucose (TyG) index, a metric derived from fasting triglyceride and blood glucose levels, had been proposed as a reliable surrogate marker of insulin resistance (5). Previous studies had reported that a higher TyG index was associated with an increased risk of cardiovascular disease (CVD) (6, 7), chronic kidney disease (8), and diabetic retinopathy (9). It had also been reported that there is a positive correlation between the TyG index and the prognosis in patients with HF and type 2 DM (10). However, the underlying mechanisms were unexplored.

Cardiovascular magnetic resonance (CMR) imaging has emerged as a novel, non-invasive diagnostic tool to assess myocardial fibrosis (11), a key pathological process in HF (12). The importance of myocardial fibrosis to predict the prognosis in different cohorts of patients had been reported (13); however, there are limited clinical data on the interaction between insulin resistance and myocardial fibrosis in patients with HF.

This study investigated the association between TyG index and myocardial fibrosis among patients with advanced HF. We also determined the possibility that TyG index and myocardial fibrosis could serve as new biomarkers for the prognosis in patients with HF.

## Methods

### Study Design and Population

We retrospective reviewed hospitalized HF patients (aged ≥18 years) with CMR examination from January 2019 to December 2020. Patients with acute myocardial infarction, history of malignancy, sepsis, severe renal function failure [estimated glomerular filtration rate (eGFR) <30 mL/min per 1.73 m^2^ or under renal replacement therapy], severe anemia (Hb <60 g/L), autoimmune disease, heart transplantation, or severe hepatic disease or without data for calculating TyG index were excluded.

The study complied with the principles of the Declaration of Helsinki and was approved by the committee of the institutional review board at Shunde Hospital, Southern Medical University, China (no. 20200320). Because of the retrospective design of the current study, the patient informed consent form was waived by the institutional review board.

### Baseline Characteristics, TyG Index Detection, and CMR Examination

We collected baseline characteristics, clinical data, and biochemistry tests from the hospital medical records. Hypertension was defined as systolic blood pressure ≥140 mm Hg and/or diastolic blood pressure ≥ 90 mm Hg or received antihypertensive treatment according to the Chinese guideline for the diagnostic and management of hypertension (14). DM was defined as fasting blood glucose (FBG) ≥7.0 mmol/L and/or hemoglobin A1_c_ (HbA1_c_) ≥6.5% or received antihyperglycemic medications (15). We calculated the eGFR according to the Modification of Diet in Renal Disease equation adapted for Chinese patients (16).

Fasting venous blood was collected, and biochemistry tests were performed in the second morning after the patients were admitted. Hemoglobin, FBG, HbA_1c_, total cholesterol (TC), triglycerides, low-density lipoprotein-C (LDL-C), high-density lipoprotein cholesterol, serum creatinine, high-sensitivity C-reactive protein (hs-CRP), and N-terminal pro–B-type natriuretic peptide (NT-proBNP) levels were detected. LDL-C was calculated using the Friedewald equation (17, 18). TyG index was calculated using the following: Ln [fasting triglycerides (mg/dL) × FBG (mg/dL)/2] (5).

Two experienced radiologists performed CMR measurements and T1 mapping during the hospitalization of the patients. Quantification of extracellular volume (ECV) fraction by T1-mapping technique in CMR imaging was used to assess myocardial fibrosis. The detailed procedure had been reported in the previous study (19). Briefly, all patients were examined in the supine position using a 3.0-T scanner (Skrya; Siemens Medical Solutions, Erlangen, Germany). A total dose of 0.1 mmol/kg gadobutrol (Gadavist, Bayer Healthcare Leverkusen, Germany) was injected at a rate of 2.0 to 3.0 mL/s; 10–15 min after contrast injection, short- and long-axis two-dimensional inversion recovery late gadolinium enhancement images were acquired to evaluate focal myocardial fibrosis. Pre- and post-contrast myocardial T1 were measured in six regions of interest in the myocardium (anterior, anterolateral, inferolateral, inferior, inferoseptal, anteroseptal) and in the left ventricular blood pool. We calculated the ECV fraction using the following formula (20), in which R1 represents 1/T1, myo pre and myo post represent the precontrast and postcontrast myocardial T1 values, respectively, and blood pre and blood post represent the precontrast and postcontrast blood pool T1 values, respectively.

$$\begin{array}{l} ECV\;fraction\;=\\ \left(1-hematocrit\right)\times \frac{R1\;myo\;post-R1\;myo\;pre}{\;R1\;blood\;post-R1\;blood\;pre} \end{array}$$

### Follow-Up and Endpoint Ascertainment

The primary outcomes in the current study were defined as composite endpoints of all-cause death or HF rehospitalization (21). Patients were followed by reviewing the electronic medical record and/or telephone interview with the participants (or the family members if patients were deceased). The follow-up duration was up to February 28, 2021.

### Statistical Analysis

All the included patients were categorized as with or without primary outcome events during follow-up. Categorical variables are presented as numbers and percentages. Continuous variables are presented as mean and standard deviation (SD), or median and interquartile range. Baseline characteristics of patients with or without primary outcome events were compared using the χ^2^ test for categorical variables, Wilcoxon rank-sum test for non–normally distributed continuous variables, and two-tailed *t*-test for normally distributed continuous variables.

Non-Gaussian data, including NT-proBNP level, left ventricular ejection fraction, eGFR, hs-CRP, ECV fraction, and cholesterol level, were log_2_-transformed. We used the Pearson product–moment correlation coefficient (*r*) as a measure of linear association between covariates and ECV fraction and performed multivariate linear regression analysis to identify factors associated with levels of ECV fraction.

Patients were divided into three groups according to tertiles of TyG index, ECV fraction, and NT-proBNP levels. Kaplan–Meier curves were employed to evaluate the study endpoints over time, and the log–rank test was used to assess differences in outcome events among different groups. Predictors of outcome were analyzed through univariate and multivariate adjusted Cox regression analyses, and the hazard ratios (HRs) and corresponding 95% confidence intervals (CIs) were presented. Risk factors with *P* < 0.10 in univariable analysis were included in the multivariable Cox regression model. Finally, receiver operating characteristic (ROC) curves and the area under the curve (AUC) were used to identify the potential relationships among TyG index, ECV fraction, and NT-proBNP levels and the primary outcomes. We used the method proposed by Delong et al. for the comparisons of the difference between two AUCs (22).

Statistical analyses were performed using SPSS version 20.0 (IBM Corp, Armonk, NY, USA). All *P*-values were two-tailed, and a *P* < 0.05 was considered statistically significant.

## Results

### Baseline Characteristics of the Included HF Patients

**Figure 1:**
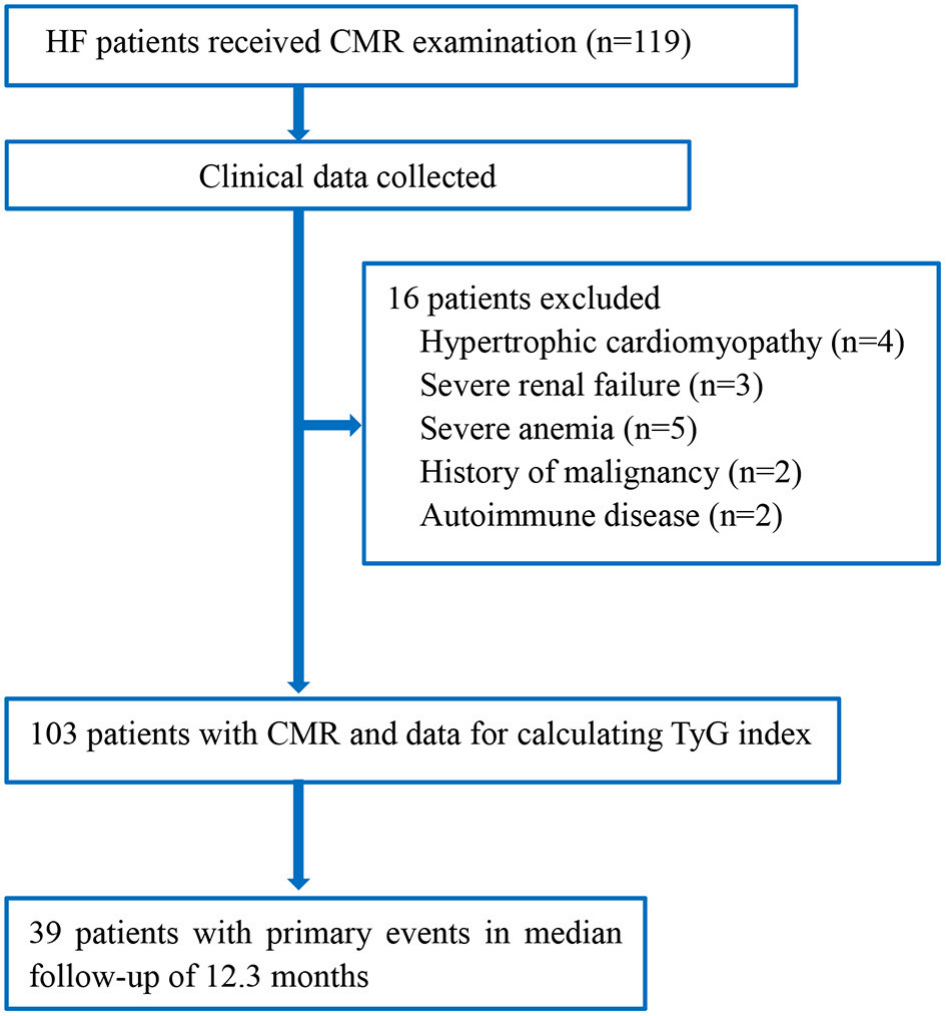
Flowchart of the study. CMR, cardiac magnetic resonance; HF, heart failure; TyG index, triglyceride–glucose index.

We screened 119 HF patients who had received CMR examination; 16 were excluded because of predefined exclusion criteria (Figure 1). Finally, 103 patients (mean age = 58.3 years) were included in the analysis. The demographic and clinical characteristics of the included patients with and without events are presented in Table 1. Compared with patients without primary adverse outcomes during follow-up, those with events had higher levels of TyG index, NT-proBNP, and ECV fraction (all *P* < 0.001).

**Table 1:** Baseline characteristics of the included HF patients.

	**All HF patients** ** (*n* = 103)**	**With events** ** (*n* = 39)**	**Without events** ** (*n* = 64)**	***P*-value**
**Clinical characteristics and comorbidities**				
Age (years)	58.3 (8.9)	57.9 (9.0)	58.6 (9.0)	0.71
Male [n (%)]	71 (68.9)	27 (69.2)	44 (68.7)	0.87
Smoking [*n* (%)]	34 (33.0)	13 (33.3)	21 (32.8)	0.87
Hypertension [*n* (%)]	71 (68.9)	31 (79.5)	41 (62.5)	0.11
Diabetes [*n* (%)]	45 (43.7)	15 (38.5)	30 (46.9)	0.53
Atrial fibrillation/flutter [n (%)]	47 (45.6)	17 (43.6)	30 (46.9)	0.90
LVEF	48.9 (38.9, 58.7)	46.7 (38.9, 58.6)	51.5 (41.2, 58.8)	0.58
**Causes of heart failure**				0.92
Ischemic heart disease	68 (66.0)	25 (64.1)	43 (67.2)	
Non-ischemic heart disease	35 (34.0)	14 (35.9)	21 (32.8)	
**Current medication**				
ACEI/ARBs [*n* (%)]	71 (68.9)	28 (71.8)	43 (67.2)	0.79
Aldosterone antagonist [*n* (%)]	73 (70.9)	27 (69.2)	46 (71.9)	0.95
CCB [*n* (%)]	33 (32.0)	13 (33.3)	20 (31.2)	0.99
β-Blockers [*n* (%)]	48 (46.6)	19 (48.7)	29 (45.3)	0.89
Loop diuretics/HCT [*n* (%)]	81 (78.6)	28 (71.8)	53 (82.8)	0.28
Digoxin [*n* (%)]	55 (53.4)	21 (53.8)	34 (53.1)	0.89
Statins [*n* (%)]	87 (84.5)	34 (87.2)	53 (82.8)	0.75
Antithrombotics [*n* (%)]	77 (74.8)	27 (69.2)	50 (78.1)	0.44
**Physical examination**				
Heart rate (beats/min)	92.2 (18.2)	92.9 (16.2)	91.7 (19.4)	0.74
Systolic BP (mm Hg)	147.6 (23.6)	146.3 (23.5)	148.4 (23.8)	0.67
Diastolic BP (mm Hg)	81.7 (16.3)	82.5 (16.4)	81.2 (16.4)	0.92
BMI (kg/m^2^)	25.5 (5.0)	25.8 (4.5)	25.3 (5.3)	0.25
**Laboratory indices**				
Hemoglobin (g/L)	115.5 (18.7)	115.8 (18.2)	115.2 (19.1)	0.88
ALT (IU/L)	34.5 (32.6, 44.7)	34.5 (32.5, 47.8)	34.9 (33.2, 44.7)	0.55
eGFR (mL/min per 1.73 m^2^)	51.8 (45.2, 74.7)	50.4 (43.6, 74.5)	52.8 (45.3, 74.5)	0.66
FPG (mmol/L)	8.3 (6.3, 10.5)	8.5 (6.6, 10.8)	7.7 (5.8, 10.3)	0.09
HbA_1c_	6.3 (5.7, 7.4)	6.4 (5.9, 7.4)	5.9 (5.6, 7.6)	0.09
TC (mmol/L)	5.0 (3.8, 5.5)	4.9 (3.9, 5.3)	5.1 (3.9, 5.7)	0.70
LDL-C (mmol/L)	2.7 (2.1, 3.0)	2.6 (2.1, 2.9)	2.9 (2.4, 3.1)	0.42
HDL-C (mmol/L)	1.0 (0.9, 1.2)	0.9 (0.8, 1.1)	1.1 (0.9, 1.2)	0.45
Triglyceride (mmol/L)	2.4 (1.9, 3.5)	2.5 (1.9, 3.6)	2.2 (1.7, 3.4)	0.18
hs-CRP (mg/L)	7.7 (2.1, 10.2)	8.5 (4.0, 14.7)	5.8 (1.6, 8.9)	0.07
Sodium (mmol/L)	133.9 (11.5)	135.4 (11.9)	133.0 (11.3)	0.30
Potassium (mmol/L)	4.2 (0.9)	4.1 (1.0)	4.3 (0.8)	0.049
NT-proBNP (ng/L)	5,723.0 (3,259.6, 8,292.9)	7,856.4 (5,802.7, 8,859.5)	4,013.7 (2,450.5, 655.2)	<0.001
ECV fraction (%)	36.5 (33.4, 39.6)	39.6 (36.2, 41.1)	35.8 (32.4, 37.9)	<0.001
TyG index	10.0 (0.82)	10.7 (0.81)	8.6 (0.78)	<0.001

*Continuous variables are presented as median (interquartile range) or mean (standard deviation). Categorical variables are expressed as number (percentages)*.

*ACEI/ARB, angiotensin-converting enzyme inhibitors or angiotensin II receptor blockers; ALT, alanine aminotransferase; BMI, body mass index; BP, blood pressure; CCB, calcium-channel blocker; ECV, extracellular volume; eGFR, estimated glomerular filtration rate; FPG, fasting plasma glucose; HbA_1c_, glycated hemoglobin; HCT, hydrochlorothiazide; HDL-C, high-density lipoprotein cholesterol; HF, heart failure; hs-CRP, high sensitive C-reactive protein; LDL-C, low-density lipoprotein cholesterol; LVEF, left ventricular ejection fraction; NT-proBNP, N-terminal pro–B-type natriuretic peptide; TyG index, triglyceride–glucose index*.

### Associations of Clinical and Laboratory Variables With ECV Fraction

Pearson correlation analysis showed that Log_2_(TyG index) (*r* = 0.34, *P* = 0.01), Log_2_(hs-CRP) (*r* = 0.32, *P* = 0.02), and Log_2_(HbA_1c_) (*r* = 0.28, *P* = 0.04) were positively associated with ECV fraction (Table 2). However, in multivariate linear regression analysis, TyG index was the only significant factor determined for ECV fraction (*r*_partial_ = 0.36, *P* = 0.01). The *R*^2^ value of SFRP2 for ECV fraction was 0.13, which indicated that 13.0% of the total ECV fraction variation can be attributed to the TyG index.

**Table 2:** Association between clinical variables and ECV fraction in HF patients.

**Variables**	**ρ**	***P*-value**
Age	−0.11	0.35
Sex	0.17	0.27
Smoking	0.05	0.77
Hypertension	0.18	0.25
Systolic blood pressure	−0.15	0.61
Ischaemic etiology	0.25	0.10
Atrial fibrillation/flutter	0.05	0.71
Log_2_ (LVEF)	−0.15	0.23
Heart rate	0.04	0.79
Body mass index	0.19	0.68
Log_2_ (ALT)	−0.04	0.65
Log_2_ (eGFR)	0.25	0.08
Log_2_ (Fasting plasma glucose)	0.26	0.07
Log_2_ (HbA_1c_)	**0.28**	**0.04**
Hemoglobin	0.06	0.82
Log_2_ (TC)	−0.20	0.34
Log_2_ (HDL-C)	0.08	0.63
Log_2_ (LDL-C)	0.15	0.42
Log_2_ (Triglyceride)	0.28	0.07
Log_2_ (hs-CRP)	**0.32**	**0.02**
Sodium	−0.06	0.67
Potassium	−0.20	0.17
Log_2_ (NT-proBNP)	0.05	0.73
TyG index	**0.34**	**0.01**

*ALT, alanine aminotransferase; ECV, extracellular volume; eGFR, estimated glomerular filtration rate; HbA_1c_, glycated hemoglobin; HDL-C, high-density lipoprotein cholesterol; HF, heart failure; hs-CRP, high sensitive C-reactive protein; LDL-C, low-density lipoprotein cholesterol; LVEF, left ventricular ejection fraction; NT-proBNP, N-terminal pro–B-type natriuretic peptide; TyG index, triglyceride–glucose index*.

*Bold values indicates data with statistical significance (P < 0.05)*.

### Prognostic Value of Markers in HF Patients

**Figure 2:**
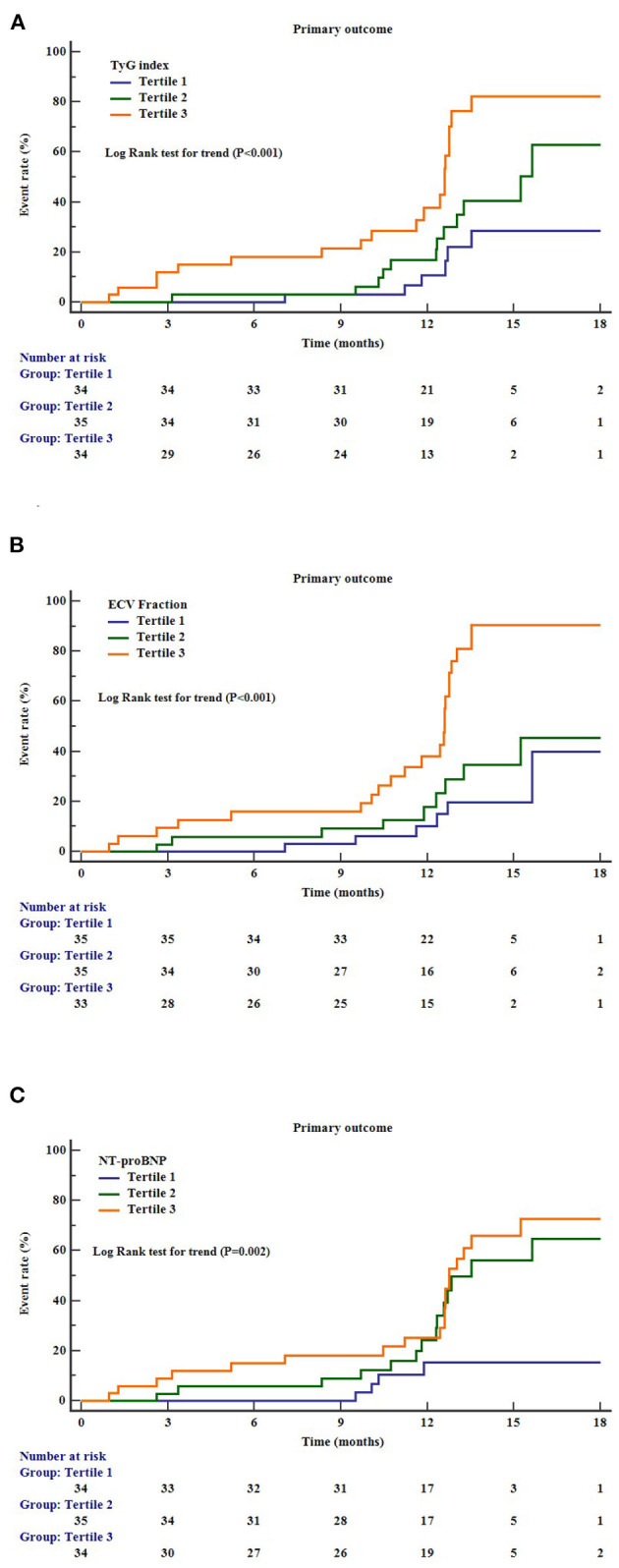
Cumulative incidence curves for the primary outcomes according to the different markers. **(A)** TyG index, triglyceride–glucose index; **(B)** ECV, extracellular volume; **(C)** NT-proBNP, N-terminal pro–B-type natriuretic peptide.

During a mean follow-up period of 12.3 months (interquartile range = 9.2–16.9 months), 39 patients (37.9%) in the cohort experienced the composite primary outcome. Kaplan–Meier survival analysis showed that higher levels of TyG index (log–rank test for trend: *P* < 0.001), ECV fraction (log–rank test for trend: *P* < 0.001), and NT-proBNP (log–rank test for trend: *P* = 0.002) were significant predictors of composite primary outcomes (Figure 2). In multivariate Cox regression models, presence of diabetes (HR = 1.28, 95% CI = 1.01–1.62), higher TyG index (HR = 2.01, 95% CI = 1.03–4.01), ECV fraction (HR = 1.73, 95% CI = 1.04–2.88), and NT-proBNP (HR = 2.13, 95% CI = 1.08–4.20) were independent risk factors for the primary outcomes (Table 3).

**Table 3:** The association between risk factors and primary outcome in HF patients.

**Variables**	**Univariate**		**Multivariate**	
	**HR (95%CI)**	***P*-value**	**HR (95%CI)**	***P* value**
Age (each 10 years)	1.10 (0.98, 1.23)	0.09		
Sex (female vs. male)	1.08 (0.14, 8.33)	0.94		
Diabetes (yes vs. no)	1.32 (1.04, 1.68)	0.02	1.28 (1.01, 1.62)	0.04
Hypertension (yes vs. no)	1.12 (0.35, 3.58)	0.85		
Smoking (yes vs. no)	1.75 (0.68, 4.50)	0.25		
BMI (each 1 kg/m^2^)	1.08 (0.55, 2.12)	0.82		
HbA_1c_ (each doubling)	1.85 (1.02, 3.36)	0.04		
Triglyceride (each doubling)	1.35 (0.75, 2.47)	0.31		
LVEF (each doubling)	0.85 (0.45, 1.61)	0.62		
eGFR (each doubling)	0.66 (0.17, 2.56)	0.55		
NT-proBNP (each doubling)	3.35 (1.36, 8.25)	0.008	2.13 (1.08, 4.20)	0.03
hs-CRP (each doubling)	1.32 (0.45, 3.87)	0.61		
TyG index (per SD increment)	2.78 (1.06, 7.29)	0.04	2.01 (1.01, 4.01)	0.047
ECV fraction (each doubling)	1.98 (1.10, 3.56)	0.02	1.73 (1.04, 2.88)	0.03

*BMI, body mass index; CI, confidence interval; ECV, extracellular volume; eGFR, estimated glomerular filtration rate; HbA_1c_, glycated hemoglobin; HR, hazard ratio; LVEF, left ventricular ejection fraction; hs-CRP, high sensitive C-reactive protein; NT-proBNP, N-terminal pro–B-type natriuretic peptide; TyG index, triglyceride–glucose index*.

**Figure 3:**
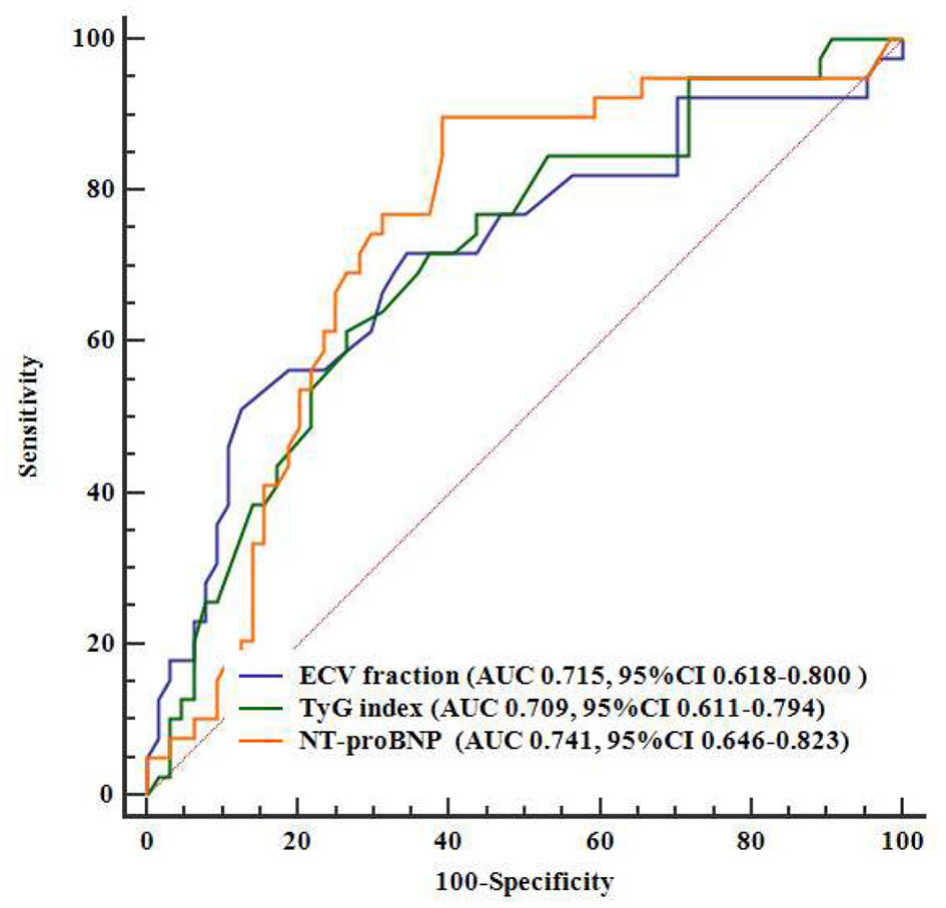
Comparisons between AUCs on ROCs. AUC, area under the curve; ECV, extracellular volume; NT-proBNP, N-terminal pro–B-type natriuretic peptide; ROC, receiver operating characteristics; TyG index, triglyceride–glucose index.

ROC analysis showed that levels of TyG index (AUC = 0.709, 95% CI = 0.611–0.794), ECV fraction (AUC 0.715, 95% CI = 0.618–0.80), and NT-proBNP (AUC 0.741, 95% CI = 0.646–0.823) had a significant predictive role on the primary outcomes (Figure 3). Pairwise comparisons of ROC curves showed that there were no significant differences in AUC among the three markers (all *P* > 0.63).

## Discussion

To the best of our knowledge, this is the first study to report that the TyG index, a surrogate marker of insulin resistance, is positively associated with myocardial fibrosis detected by CMR. We also found that a higher TyG index was associated with poorer prognosis in HF patients after adjusting for other risk factors, including DM.

Myocardial fibrosis is a key pathological process and represents a potential therapeutic target for HF (23). The gold standard for evaluating myocardial fibrosis is myocardial biopsy. However, the invasive nature limits its clinical use. In the past decade, studies have demonstrated myocardial fibrosis estimated by CMR can provide important prognostic value (13, 23). In this study, we also found that a higher ECV fraction was associated with an increased adverse outcome in patients with HF, further supporting the notion that ECV fraction could be used as a non-invasive marker to evaluate myocardial fibrosis and risk stratification. Furthermore, we found that the TyG index was the only significant factor determined for ECV fraction, which provided the message that insulin resistance is an important risk factor for myocardial fibrosis. Our study was supported by another retrospective cohort study, which showed a positive correlation between the TyG index and the prognosis of patients with HF and type 2 DM (10). Several mechanisms involve the interaction among insulin resistance, myocardial fibrosis, and worse prognosis in HF. First, insulin resistance is associated with low-degree inflammation, which plays an important role in the pathogenesis of cardiomyocyte apoptosis and myocardial fibrosis (24). Second, insulin resistance has been associated with the increased sympathetic nervous system and renin–angiotensin–aldosterone system activity; both of them were involved in myocardial fibrosis and cardiac dysfunction (25, 26). Third, insulin resistance is related to the deposition of extracellular matrix deposition (27) and intramyocardial lipids, (28) resulting in subsequent myocardial fibrosis.

The homeostatic model assessment of insulin resistance (HOMA-IR) is another marker broadly used for defining insulin resistance. Because of the retrospective design, HOMA-IR data were not available in the current study. However, several studies aimed to compare the predictive effect of the TyG index and HOMA-IR for cardiovascular risk. In a cross-sectional study, the TyG index was still significantly associated with coronary artery stenosis in patients with type 2 DM (29). It had been reported that the TyG index was independently associated with arterial stiffness and 10-year CVD risk in a Chinese cohort, while in the same cohort, the association of the HOMA-IR and the 10-year CVD risk was absent when adjusting for multiple risk factors (6). Other studies also showed that the TyG index was more independently associated with increased arterial stiffness and coronary artery calcification than HOMA-IR in Korean (30, 31). Nowadays, the detection of metrics for calculating the TyG index (including fasting triglycerides and FBG) is convenient and affordable worldwide. However, the calculation for HOMA-IR was based on fasting insulin, which was much expensive than triglycerides and not available in most clinical laboratories. Therefore, we proposed that the TyG index could be considered as a more convenient marker of insulin resistance and regarded as a useful predictor of adverse prognosis in HF.

Some limitations to this study should be noted. First, the retrospective design of the current research would cause recall bias, and residual confounders could not be totally avoided. Prospective cohort studies would be useful to further ascertain the association between TyG index and myocardial fibrosis. Second, our study had a relatively small sample size, which limits us to further perform analysis based on patients with and without DM. However, in the multivariable Cox regression analysis, the association between TyG index and primary outcome events was still significant after adjusting for DM, which indicated that the higher TyG index was associated with a worse prognosis of HF independent of DM. Third, the TyG index was available only at baseline, not during follow-up. Thus, any changes in the TyG index that may have occurred in response to treatment of HF are unknown and require further exploration. Finally, the ECV fraction is a surrogate marker, not the gold standard of myocardial fibrosis. Therefore, the association between TyG index and ECV fraction is only an indirect evidence of myocardial fibrosis.

## Conclusions

TyG index is a novel biomarker of myocardial fibrosis in HF patients. We also demonstrated that a higher TyG index was significantly associated with a worse prognosis in HF, which can be considered as a useful risk stratification metric in the management of HF.

## Data Availability Statement

The raw data supporting the conclusions of this article will be made available by the authors, without undue reservation.

## Ethics Statement

The studies involving human participants were reviewed and approved by Institutional review board at Shunde Hospital, Southern Medical University, China. Written informed consent for participation was not required for this study in accordance with the national legislation and the institutional requirements.

## Author Contributions

SY, YD, and HC: conception, design, and administrative support. SY, YD, ZL, and RZ: data analysis and interpretation. SY, YD, ZL, and XL: manuscript writing. All authors provision of study materials or patients, collection and assembly of data, and final approval of manuscript.

## Conflict of Interest

The authors declare that the research was conducted in the absence of any commercial or financial relationships that could be construed as a potential conflict of interest.
